# Veterinary Medical Association of New Jersey

**Published:** 1894-01

**Authors:** S. Lockwood

**Affiliations:** Secretary


### SOCIETY PROCEEDINGS.

### VETERINARY MEDICAL ASSOCIATION OF NEW JERSEY.

The thirtieth regular meeting of the Veterinary Medical Association of New Jersey was held at the U. S. Hotel, in Morristown, on Thursday, Dec. 14th, 1893.

Dr. Gerth, the president, being absent, the meeting was called to order by Dr. Drummond of Woodbridge, N. J., second vice-president.

On call of the roll, the following members answered to their names: Drs. W. B. E. Miller, Camden, N. J.; J. W. Hawk, Newark, N. J.; James C. Dustan, Morristown, N. J.; B. F. King, Little Silver, N. J.; W. Runge, Newark, N. J.; R. O. Hasbrouck, Passaic, N. J.; R. R. Letts, Hoboken, N. J.; J. M. Everetts, Hackettstown, N. J.; M. M. Stage, Dover, N. J.; A. W. Axford, Naughreght, N. J.; H. Exton, Washington, N. J.; B. L. Drummond, Woodbridge; S. Lockwood, Woodbridge, N. J.; Ira Kilburn, South Orange, N. J.; Prof. Leonard Pearson, Philadelphia, Pa. Delegates from Pennsylvania State Association were present, also: Dr. J. W. Stickler, M. D., Orange, N. J.; Dr. A. V. Baldwin, M. D., New Brunswick, N. J.; Dr. G. Swain, M. D., Chatham, N. J.; and about twenty visitors, stockgrowers, dairymen and farmers, who were interested in the question of tuberculosis, which was one of the special orders of this meeting.

The regular order of business was first gone over, and an interesting and instructive paper on influenza in the horse was read by *Dr. Miller*, of Camden.

*Prof. Pearson*, of the University of Pennsylvania, read an interesting paper on tuberculosis and the use of tuberculine in diagnosing the disease. He gave a history of a number of experiments with the same, also of a number of experiments on pigs and rabbits. He also spoke of the dangers of tuberculosis and the necessity of both State and National legislation for this disease.

*Dr. Stickler*, of Orange, followed with some very interesting and instructive remarks on the disease, and gave an alarming report of the number of deaths from the disease in the human family in the last ten years in this State, averaging over three thousand each year. He also urged the necessity for better and more effective legislation.

*Dr. Baldwin*, of New Brunswick, followed with other pertinent remarks and figures. He also gave an interesting account of a number of experiments that he had seen and assisted in at the Experimental Station, near New Brunswick. He, too, urged the need of proper legislation. Remarks were also made by Drs. Hawk, Miller, Dustan, Lockwood, Swain and others, all pertaining to the subject.

A characteristic specimen of the disease was presented before the Association by Dr. Hawk, in a pair of lungs from a registered Jersey cow, owned near Orange, N. J., which he had slaughtered a few days ago, and which had been kept as a private family cow. The lungs were a solid mass of tubercles throughout.

*Dr. Stickler* requested the Association to appoint a committee to confer with a committee from the State Medical Association and other associations, for the purpose of formulating a bill to be presented to the next legislature, having for its object the eradication of the disease from the dairy herds known to be infected in various parts of the State, and to prevent the sale of milk from such herds.

The Association also adopted resolutions of condolence with the family of the Hon. J. M. Rusk, deceased, late Secretary of Agriculture, who was an honorary member of the Association.

*Resolutions* were also passed approving the action of the late International Veterinary Congress, held in Chicago, Ill., and of the resolutions adopted there relating to the spoils system in the veterinary appointments recently made by the Bureau of Animal Industry.

*A resolution* was also passed disapproving the action of the officers of the National Veterinary College at Washington, in making their course one or two years instead of a more lengthy one, believing it prejudicial to the best interests of veterinary science, to graduate students after such a short course of study.

*Drs. Dustan, Letts and Drummond* were appointed essayists for the next regular annual meeting, which will be held in Trenton, on the second Thursday in April, 1894.

Some interesting cases were then reported by some of the members, and thanks were then extended to the visitors and others for their attendance and interesting remarks made; also to mine host A. E. Voorhees for the use of his elegant parlors in which to hold the meetings; after which the meeting adjourned.

S. LOCKWOOD, *Secretary.*

---

